# Perceived Familial Financial Insecurity and Obesity Among Korean Adolescents During the COVID-19 Pandemic

**DOI:** 10.2188/jea.JE20240038

**Published:** 2024-12-05

**Authors:** Fumie Kaneko, Eunji Kim, Hokyou Lee, Kokoro Shirai, Ryo Kawasaki, Hyeon Chang Kim

**Affiliations:** 1 Department of Public Health, Yonsei University Graduate School, Seoul, South Korea; 2 Division of Public Health, Department of Social Medicine, Osaka University Graduate School of Medicine, Osaka, Japan; 3 Department of Preventive Medicine, Yonsei University College of Medicine, Seoul, South Korea; 4 Artificial Intelligence Center for Medical Research and Application, Osaka University Hospital, Osaka, Japan; 5 Institute for Innovation in Digital Healthcare, Yonsei University, Seoul, South Korea

**Keywords:** obesity, adolescent, financial insecurity, socioeconomic status, recession

## Abstract

**Background:**

In high-income countries, socioeconomically disadvantaged adolescents experience a higher risk of obesity, which may have been further exacerbated during the early phase of the coronavirus disease 2019 (COVID-19) pandemic. This study aimed to investigate the association between obesity and familial financial insecurity, utilizing data on subjective household socioeconomic status (SES) and perceived family-level financial deterioration induced by COVID-19.

**Methods:**

We utilized data from the Korea Youth Risk Behavior Survey, a nationally representative sample of Korean adolescents, in 2020 and 2021. The independent and joint associations of two primary exposures, subjective household SES and perceived family-level financial deterioration, with obesity were assessed using multivariable logistic regression models.

**Results:**

Among 106,979 adolescents aged 12–18 years, 16.9% of boys and 9.0% of girls met the criteria for obesity. Notably, 70.5% reported experiencing COVID-19-related financial deterioration. Both subjective household SES and perceived family-level financial deterioration independently and synergistically increased the odds of obesity. A graded association was observed between obesity and lower SES and more severe financial deterioration, particularly among girls. Younger adolescents were more sensitive to household SES, whereas older adolescents were more sensitive to financial deterioration.

**Conclusion:**

While the COVID-19 pandemic presented a unique social context, our findings highlight that financially insecure adolescents were at an increased risk of obesity during the early phase of the pandemic. This underscores the need for obesity-prevention strategies in times of macroeconomic recession to address not only the persistent influence of household SES but also the direct and indirect effects of family-level financial deterioration.

## INTRODUCTION

Obesity among children and adolescents is an emerging public health and medical problem.^1^^,^^2^ The prevalence of adolescent obesity has been increasing worldwide,^3^ including in Korea,^4^ and obesity in adolescence is a significant risk factor for chronic diseases.^1^^,^^4^^,^^5^ Obesity-related cardiovascular risk factors, such as dyslipidemia and high blood pressure, begin to develop in early childhood and progress to measurable vascular changes in young adulthood.^5^^,^^6^

Children from financially insecure families may be at higher risk of obesity during a macroeconomic recession.^7^^–^^9^ Although the magnitude varies between countries and social contexts, a lower socioeconomic status (SES) is associated with a higher risk of obesity among adolescents and adults in high-income countries.^10^^,^^11^ In contrast, the effect of family-level financial deterioration during a macroeconomic recession on adolescent obesity is unclear. The coronavirus disease 2019 (COVID-19) pandemic has affected both the global economy and domestic economy of Korea,^12^^,^^13^ as evidence by ongoing investigations into its health-related impacts has revealed. In this study, we aimed to examine the independent and joint associations of subjective household SES and perceived financial deterioration induced by COVID-19 with obesity among Korean adolescents.

## METHODS

### Study population

We conducted an analysis using serial cross-sectional data from the combined 2020 and 2021 waves of the Korean Youth Risk Behavior Survey (KYRBS). The KYRBS is an annual, nationwide, school-based web survey, conducted to examine health-risk behaviors among middle and high school students aged 12–18 years. The survey uses multistage cluster sampling, designed to include 400 middle schools and 400 high schools. A detailed description of this process is available elsewhere.^14^ The survey was conducted from August to November, with 54,948 students (94.9% response rate) in 2020 and 54,848 (92.9% response rate) in 2021. The study participants in 2020 and 2021 did not overlap. After excluding 2,817 participants (1,414 in 2020 and 1,403 in 2021) for whom information on height or weight was missing, 106,979 were included in the primary analysis. Further analysis was conducted to examine the influence of parental characteristics among the participants who consented to participate in the household survey (*N* = 76,900) (eFigure 1).

### Measurements

One of the two primary exposures in this study was subjective household SES, which was obtained using a self-report questionnaire. Each student was asked to rate their household SES as high, upper-middle, middle, lower-middle, and low, which we re-categorized into three categories: higher (high and upper-middle), middle, and lower (lower-middle and low). The other primary exposure variable of interest was the perceived financial deterioration attributable to the COVID-19 pandemic. Participants were asked to characterize the financial impact of the COVID-19 pandemic on their household as severe, moderate, minimal, or none. Pertinent data relating to parental background were obtained after acquiring the requisite permission for continued household surveys. These included paternal and maternal educational attainment, delineated as higher education (university level or higher), secondary education (high school level or lower), or not known. Furthermore, details regarding the parents’ living arrangements were collated, with classifications including cohabitation with both parents, only the father, only the mother, or neither parent.

Participants reported their height and weight as part of the survey questionnaire, from which body mass index (BMI) was computed. The computed BMI was transposed into sex- and age-specific percentiles in alignment with the 2017 Korean National Growth Charts for children and adolescents.^15^ Individuals were classified as having obesity if their BMI was in the 95th percentile or higher.^16^

Demographic information, including sex, age (represented by school grade), type of living arrangement (residing with family, with relatives, or in a boarding house, dormitory, or childcare facility), urbanicity (metropolitan city, medium-sized or small city, or rural area), and school type (co-educational or single-sex school), was self-disclosed by the participants.

### Statistical analysis

In all analyses, boys and girls were analyzed separately, as sex differences have been suggested in previous studies.^11^ First, descriptive statistics, in the form of means, standard deviations (SDs), and percentages, were calculated. Multiple logistic regression analysis was conducted to estimate the association between the exposure variables and obesity as a binary outcome. Odds ratios (ORs) and 95% confidence intervals (CIs) were calculated for two models^17^: model 1, adjusted for either of two exposures and sociodemographic information (age, living arrangement, and urbanicity); and model 2, adjusted for both exposures and sociodemographic variables. The sociodemographic variables incorporated in the models have previously been reported to be associated with BMI and possibly with SES or COVID-19-related financial deterioration.

A stratified analysis according to three socioeconomic strata was conducted to assess effect modification. Statistical interaction was confirmed using interaction terms of SES and financial deterioration. We examined the joint association between the two exposures by creating a 12-category exposure (ie, the combination of the three familial SES categories and four financial deterioration categories) and obesity. In all logistic regression models, middle- and high-school students were analyzed separately.

An exploratory analysis was conducted with the participants who agreed to participate in the household survey to assess further background factors. The models for this analysis were further adjusted for parental variables and school type (co-educational or single sex).

All the analyses were performed using SAS version 9.4 (SAS Institute Inc., Cary, NC, USA). As the KYRBS was designed for use with weighted values derived using stratified, clustered, or multistage sampling methods, we used the “proc surveylogistic” procedure in all logistic regression models. A two-sided *P* < 0.05 was considered statistically significant.

### Ethical considerations

The study protocol for the secondary data analysis was approved by the Institutional Review Board of Severance Hospital, Yonsei University Health System (approval no. 4-2023-0498). Informed consent was not required, as the de-identified dataset is publicly available upon registration on the survey website.

## RESULTS

### General characteristics

The mean age of the participants included in this study was 15.1 years, and 48.2% were girls (Table 1). Overall, 16.9% of boys and 9.0% of girls were classified as having obesity. Approximately half of the participants indicated their subjective household SES as middle, and approximately 70% of participants indicated that they had experienced COVID-19-related financial deterioration. The degrees of perceived financial deterioration classified as severe, moderate, and minimal were distributed as 6.3%, 24.4%, and 38.8%, respectively, among boys and 5.0%, 24.8%, and 41.7%, respectively, among girls. The distribution of their subjective household SES according to the original five categories is summarized in eTable 1.

**Table 1.** General characteristics of participants

Variables	Boys						Girls					
	
	Total (*n* = 55,460)		2020 (*n* = 27,687)		2021 (*n* = 27,773)		Total (*n* = 51,519)		2020 (*n* = 25,847)		2021 (*n* = 25,672)	
Age, years, mean (SD)	15.1	(1.7)	15.1	(1.8)	15.1	(1.7)	15.1	(1.8)	15.1	(1.8)	15.1	(1.8)
Obese, *n* (%)	9,369	(16.9)	4,435	(16.0)	4,934	(17.8)	4,638	(9.0)	2,244	(8.7)	2,394	(9.3)
School stage, *n* (%)												
Middle school	29,815	(53.8)	14,536	(52.5)	15,279	(55.0)	27,796	(54.0)	13,756	(53.2)	14,040	(54.7)
High school	25,645	(46.2)	13,151	(47.5)	12,494	(45.0)	23,723	(46.0)	12,091	(46.8)	11,632	(45.3)
Household socioeconomic status, *n* (%)												
Higher	22,967	(41.4)	11,388	(41.1)	11,579	(41.7)	19,023	(36.9)	9,507	(36.8)	9,516	(37.1)
Middle	25,775	(46.5)	12,727	(46.0)	13,048	(47.0)	26,401	(51.2)	13,011	(50.3)	13,390	(52.2)
Lower	6,718	(12.1)	3,572	(12.9)	3,146	(11.3)	6,095	(11.8)	3,329	(12.9)	2,766	(10.8)
COVID-19-related financial deterioration, *n* (%)												
None	16,896	(30.5)	8,480	(30.6)	8,416	(30.3)	14,668	(28.5)	7,443	(28.8)	7,225	(28.1)
Minimal	21,525	(38.8)	10,775	(38.9)	10,750	(38.7)	21,476	(41.7)	10,575	(40.9)	10,901	(42.5)
Moderate	13,524	(24.4)	6,672	(24.1)	6,852	(24.7)	12,792	(24.8)	6,517	(25.2)	6,275	(24.4)
Severe	3,515	(6.3)	1,760	(6.4)	1,755	(6.3)	2,583	(5.0)	1,312	(5.1)	1,271	(5.0)
Urbanicity, *n* (%)												
Metropolitan city	24,307	(43.8)	12,268	(44.3)	12,039	(43.3)	22,091	(42.9)	10,790	(41.7)	11,301	(44.0)
Medium-sized or small city	26,823	(48.4)	13,253	(47.9)	13,570	(48.9)	25,578	(49.6)	13,031	(50.4)	12,547	(48.9)
Rural area	4,330	(7.8)	2,166	(7.8)	2,164	(7.8)	3,850	(7.5)	2,026	(7.8)	1,824	(7.1)
Living arrangement, *n* (%)												
Living with family	52,745	(95.1)	26,284	(94.9)	26,461	(95.3)	49,476	(96.0)	24,764	(95.8)	24,712	(96.3)
Living with relatives	248	(0.4)	113	(0.4)	135	(0.5)	238	(0.5)	129	(0.5)	109	(0.4)
Other	2,467	(4.4)	1,290	(4.7)	1,177	(4.2)	1,805	(3.5)	954	(3.7)	851	(3.3)
School type, *n* (%)												
Co-educational school	37,920	(68.4)	18,592	(67.2)	19,328	(69.6)	34,156	(66.3)	17,054	(66.0)	17,102	(66.6)
All-male or all-female school	17,540	(31.6)	9,095	(32.8)	8,445	(30.4)	17,363	(33.7)	8,793	(3.0)	8,570	(33.4)

COVID-19, coronavirus disease 2019; SD, standard deviation.

### Perceived household financial insecurity and adolescent obesity

In the separate regression models for subjective household SES and perceived COVID-19-related financial deterioration (Table 2 and eTable 2), obesity was more prevalent among participants with a lower SES than among those with a higher SES, both among boys and girls. A gradient increase in ORs was observed with a lower SES and more severe financial deterioration. The association between lower SES and obesity was more significant among middle-school students than among high-school students. Additionally, the association between perceived financial deterioration and obesity was perceptible among high-school boys and middle- and high-school girls. However, this association was absent among middle-school boys.

**Table 2.** Association between household socioeconomic characteristics and obesity among adolescents

Household socioeconomic characteristics	Boys							Girls						
	
	Number of adolescents	Number (%) of individuals with obesity		Odds ratio (95% CI)				Number of adolescents	Number (%) of individuals with obesity		Odds ratio (95% CI)			
	
				Model 1^a^		Model 2^b^					Model 1^a^		Model 2^b^	
**Overall**														
Household socioeconomic status														
Higher	22,967	3,726	(16.2)	1.00	(Reference)	1.00	(Reference)	19,023	1,389	(7.3)	1.00	(Reference)	1.00	(Reference)
Middle	25,775	4,363	(16.9)	1.05	(1.00–1.11)	1.03	(0.98–1.09)	26,401	2,415	(9.2)	1.19	(1.11–1.28)	1.13	(1.06–1.22)
Lower	6,718	1,280	(19.1)	1.22	(1.13–1.31)	1.15	(1.07–1.24)	6,095	834	(13.7)	1.85	(1.69–2.03)	1.62	(1.46–1.78)
COVID-19-related financial deterioration														
None	16,896	2,671	(15.8)	1.00	(Reference)	1.00	(Reference)	14,668	1,076	(7.3)	1.00	(Reference)	1.00	(Reference)
Minimal	21,525	3,568	(16.6)	1.05	(0.99–1.12)	1.04	(0.98–1.11)	21,476	1,847	(8.6)	1.14	(1.05–1.24)	1.08	(1.00–1.18)
Moderate	13,524	2,451	(18.1)	1.18	(1.11–1.26)	1.15	(1.08–1.23)	12,792	1,363	(10.7)	1.42	(1.30–1.55)	1.27	(1.16–1.39)
Severe	3,515	679	(19.3)	1.25	(1.13–1.38)	1.20	(1.08–1.33)	2,583	352	(13.6)	1.93	(1.69–2.20)	1.58	(1.38–1.82)
**Middle-school students**														
Household socioeconomic status														
Higher	13,708	2,189	(16.0)	1.00	(Reference)	1.00	(Reference)	9,704	682	(5.9)	1.00	(Reference)	1.00	(Reference)
Middle	13,282	2,199	(16.6)	1.03	(0.96–1.10)	1.02	(0.95–1.10)	10,898	1,041	(7.7)	1.32	(1.19–1.46)	1.29	(1.16–1.43)
Lower	2,825	540	(19.1)	1.28	(1.16–1.42)	1.24	(1.12–1.38)	1,921	277	(10.5)	1.90	(1.63–2.21)	1.70	(1.44–2.01)
COVID-19-related financial deterioration														
None	9,464	1,505	(15.9)	1.00	(Reference)	1.00	(Reference)	8,540	543	(6.4)	1.00	(Reference)	1.00	(Reference)
Minimal	11,495	1,845	(16.1)	1.01	(0.93–1.09)	1.00	(0.92–1.08)	11,461	785	(6.9)	1.07	(0.96–1.19)	0.99	(0.89–1.11)
Moderate	7,074	1,241	(17.5)	1.10	(1.01–1.21)	1.07	(0.97–1.18)	6,560	540	(8.2)	1.31	(1.16–1.49)	1.15	(1.01–1.31)
Severe	1,782	337	(18.9)	1.19	(1.04–1.37)	1.12	(0.98–1.29)	1,235	132	(10.7)	1.81	(1.49–2.20)	1.48	(1.19–1.83)
**High-school students**														
Household socioeconomic status														
Higher	9,259	1,537	(16.6)	1.00	(Reference)	1.00	(Reference)	7,475	707	(9.5)	1.00	(Reference)	1.00	(Reference)
Middle	12,493	2,164	(17.3)	1.07	(1.00–1.16)	1.04	(0.96–1.12)	12,788	1,374	(10.7)	1.11	(1.01–1.21)	1.03	(0.94–1.13)
Lower	3,893	740	(19.0)	1.19	(1.07–1.31)	1.09	(0.98–1.21)	3,460	557	(16.1)	1.80	(1.60–2.02)	1.53	(1.35–1.72)
COVID-19-related financial deterioration														
None	7,432	1,166	(15.7)	1.00	(Reference)	1.00	(Reference)	6,128	533	(8.7)	1.00	(Reference)	1.00	(Reference)
Minimal	10,030	1,723	(17.2)	1.11	(1.01–1.21)	1.09	(1.00–1.19)	10,015	1,062	(10.6)	1.21	(1.07–1.36)	1.16	(1.03–1.72)
Moderate	6,450	1,210	(18.8)	1.27	(1.16–1.39)	1.09	(1.13–1.36)	6,232	823	(13.2)	1.51	(1.34–1.70)	1.37	(1.21–1.55)
Severe	1,733	342	(19.7)	1.31	(1.23–1.52)	1.27	(1.09–1.48)	1,348	220	(16.3)	2.03	(1.70–2.42)	1.69	(1.41–2.03)

CI, confidence interval; COVID-19, coronavirus disease 2019.

^a^Model 1: Adjusted for age, living arrangement, and urbanicity.

^b^Model 2: Adjusted for age, living arrangement, urbanicity, household socioeconomic status, and COVID-19-related financial deterioration.

**Figure 1.**
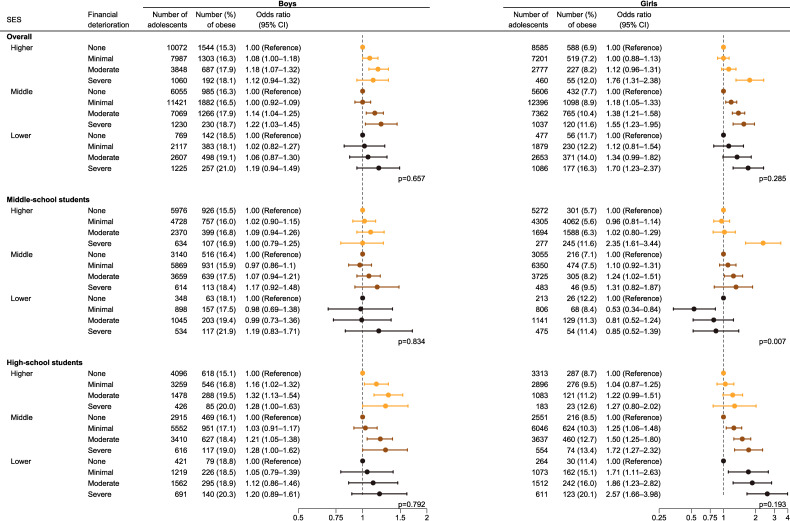
Association between COVID-19-related financial deterioration and obesity stratified according to household SES

A common pattern emerged in the analysis stratified by SES, revealing an escalation of the OR in line with the severity of perceived financial deterioration across most strata. The most potent association was observed among high-school girls in both the middle- and lower-SES categories (Figure 1). The hypothesized interaction between the subjective household SES and perceived financial deterioration in influencing the risk of obesity was not statistically significant in most subgroups (Figure 1). When middle- and high-school students were pooled, the *P*-value for interaction was 0.657 for boys and 0.285 for girls. When data of boys and girls were pooled, the *P*-value for interaction was 0.911 for middle-school students and 0.765 for high-school students.

**Figure 2.**
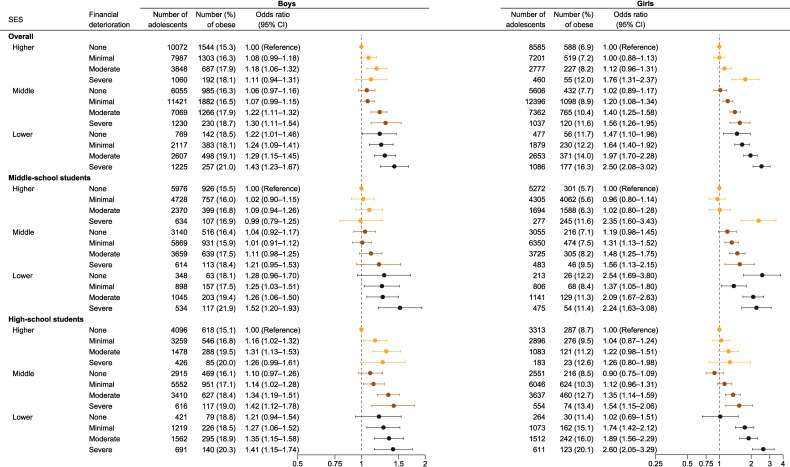
Joint association of household SES and COVID-19-related financial deterioration with obesity

Upon assessing the joint associations, we observed diverse patterns and magnitudes of ORs among the subgroups (Figure 2). Compared with the most secure group (ie, individuals with a higher SES and no financial deterioration), the lower SES groups among both middle-school boys and girls exhibited higher ORs. Among middle-school boys, the ORs did not differ according to financial deterioration within the higher-SES group. However, among middle-school girls, we observed escalated ORs in the higher-SES group with severe financial deterioration. Among high-school boys, the ORs increased both with lower SES and with financial deterioration. In contrast, in high-school girls, a lower SES intensified the gradient of elevated ORs associated with financial deterioration.

### Other sociodemographic factors and adolescent obesity

For this analysis, we included 76,900 participants, comprising 37,025 boys and 39,875 girls (participant characteristics are summarized in eTable 3). Lower paternal educational attainment was associated with obesity in all subgroups. Conversely, maternal education significantly increased the odds of obesity only among girls. A significantly heightened risk was also observed among adolescents residing with their fathers but not their mothers, except for middle-school boys. Furthermore, obesity was more prevalent among students in single-sex schools than among those in co-educational schools (Table 3).

**Table 3.** Sociodemographic factors associated with adolescent obesity

Sociodemographic factors	Boys				Girls			
	
	Middle school		High school		Middle school		High school	
			
	Adjusted^a^ OR	(95% CI)	Adjusted^a^ OR	(95% CI)	Adjusted^a^ OR	(95% CI)	Adjusted^a^ OR	(95% CI)
Household socioeconomic status								
Higher	1.00	(Reference)	1.00	(Reference)	1.00	(Reference)	1.00	(Reference)
Middle	0.96	(0.89–1.05)	0.94	(0.85–1.04)	1.20	(1.07–1.35)	0.93	(0.84–1.04)
Lower	1.16	(1.02–1.33)	1.00	(0.88–1.15)	1.30	(1.07–1.58)	1.16	(1.00–1.35)
COVID-19-related financial deterioration								
None	1.00	(Reference)	1.00	(Reference)	1.00	(Reference)	1.00	(Reference)
Minimal	0.95	(0.87–1.04)	1.13	(1.02–1.26)	0.97	(0.85–1.11)	1.13	(0.99–1.29)
Moderate	0.98	(0.88–1.09)	1.26	(1.11–1.42)	1.05	(0.90–1.22)	1.31	(1.14–1.51)
Severe	0.96	(0.81–1.14)	1.24	(1.02–1.51)	1.45	(1.13–1.85)	1.48	(1.19–1.85)
Paternal educational attainment								
University or higher	1.00	(Reference)	1.00	(Reference)	1.00	(Reference)	1.00	(Reference)
High school or lower	1.37	(1.22–1.53)	1.20	(1.09–1.50)	1.31	(1.11–1.55)	1.36	(1.21–1.53)
Not known	1.17	(1.03–1.32)	1.28	(1.09–1.51)	1.16	(0.95–1.41)	1.28	(1.07–1.54)
Maternal educational attainment								
University or higher	1.00	(Reference)	1.00	(Reference)	1.00	(Reference)	1.00	(Reference)
High school or lower	1.04	(0.93–1.17)	1.09	(0.98–1.22)	1.56	(1.34–1.82)	1.16	(1.02–1.33)
Not known	1.16	(1.02–1.31)	1.01	(0.85–1.20)	1.44	(1.16–1.78)	1.17	(0.95–1.44)
Parental cohabitation								
Father and mother	1.00	(Reference)	1.00	(Reference)	1.00	(Reference)	1.00	(Reference)
Only father	1.03	(0.80–1.33)	1.40	(1.10–1.78)	1.46	(1.04–2.06)	1.50	(1.14–1.98)
Only mother	1.28	(1.10–1.48)	1.01	(0.86–1.18)	1.06	(0.85–1.31)	1.20	(1.01–1.41)
Neither	0.92	(0.53–1.57)	1.20	(0.82–1.76)	1.29	(0.64–2.63)	1.17	(0.76–1.75)
Urbanicity								
Metropolitan city	1.00	(Reference)	1.00	(Reference)	1.00	(Reference)	1.00	(Reference)
Medium-sized or small city	0.99	(0.91–1.07)	0.92	(0.84–1.01)	1.03	(0.92–1.15)	1.06	(0.96–1.17)
Rural area	1.08	(0.93–1.27)	1.03	(0.88–1.20)	1.32	(1.07–1.62)	1.10	(0.92–1.32)
School type								
Co-educational school	1.00	(Reference)	1.00	(Reference)	1.00	(Reference)	1.00	(Reference)
All-male or all-female school	1.28	(1.17–1.41)	1.14	(1.04–1.25)	1.29	(1.13–1.46)	1.25	(1.14–1.38)

CI, confidence interval; OR, odds ratio; COVID-19, coronavirus disease 2019.

^a^Adjusted for age, living arrangement, and all variables listed above.

## DISCUSSION

In this comprehensive, nationwide study of middle- and high-school students, subjective household SES and perceived family-level financial deterioration attributable to the COVID-19 pandemic were either independently or jointly linked to an increased probability of obesity. This connection varied according to school stage and sex. The risk of obesity appeared to follow a gradient pattern. A lower SES and more severe financial deterioration yielded higher odds of obesity, especially among girls. The overall influence of subjective SES was more potent among middle-school students of both sexes, whereas perceived COVID-19-related financial deterioration had a more significant impact on high-school students. Crucially, the joint effect of subjective SES and perceived financial deterioration was additive, although no statistically significant interaction was observed in most subgroups.

Although SES is a significant risk factor for obesity at every stage of life, research on family-level financial deterioration during macroeconomic downturns has been limited to pre-adolescents and adults.^7^^–^^9^^,^^18^ Our results are consistent with those of studies of the impact of the Great Recession in 2008 on pre-adolescents, including a cross-sectional study of Portuguese children aged 6–11 years,^6^ a longitudinal cohort study of Japanese children followed up to 2011 (when they were 10 years old),^8^ and aggregated data of children in the United States.^9^ Sex-related differences were also consistent with those of previous studies, with higher odds of obesity confirmed among girls.^7^^,^^8^

The factors contributing to adolescent obesity may be multifaceted, encompassing individual biological predispositions, demographics, socioeconomic attributes, sociocultural characteristics, and lifestyle choices.^19^ The limited affordability of a healthy lifestyle is generally acknowledged to mediate the relationship between a lower SES and a higher risk of obesity.^18^ For example, maternal educational attainment affects dietary patterns,^20^ limited financial resources lead to higher consumption of low-priced but high-energy-intensity foods,^21^ and parents’ inactive lifestyles possibly account for children and adolescents engaging in less physical activity, such as leisure or extracurricular sports activities.^22^ In our exploratory analysis, parental educational attainment, parental cohabitation, and attendance at single-sex schools contributed to an increased risk of obesity. A father’s educational attainment may directly precipitate financial insecurity, thereby affecting household SES. In contrast, a mother’s education may indirectly shape her adolescent children’s lifestyle habits, such as dietary preferences, and marginally influence the family’s SES. Although the parameter of parental cohabitation did not unambiguously indicate whether two parents or a single parent led a household, it may influence the accessibility to healthy lifestyles and be partially connected to financial insecurity. Additionally, the weight of each parent, particularly the mother, reportedly correlates with the weight of their adolescent children in South Korea, irrespective of sex.^23^ Given that socioeconomic disparities in obesity among Korean adults have been predominantly limited to women,^24^ the increased risk of being overweight may be perpetuated across generations through a multitude of conduits.

In our study, the hypothesized interaction of subjective SES and perceived financial deterioration with obesity was not statistically significant in most subgroups. However, we confirmed an additive association, and the impact of subjective SES and perceived financial deterioration varied in terms of magnitude and pattern across subgroups (Figure 2 and stratified by survey year in eFigure 2). In the analysis stratified according to SES (Figure 1), the effect modification by SES was not significant, except for that among middle-school girls. Regarding the different patterns of ORs observed among middle-school girls, at least two points can be noted: first, the reference group was relatively small, and second, a lower SES had a notable impact in this group (Figure 2). Overall, subjective household SES had a more dominant effect on obesity among middle-school students, whereas financial deterioration had a more substantial influence among high-school students. These results suggest that, even within the adolescent population, heterogeneity may exist in terms of the impact of parental SES. Younger adolescents may depend more on their guardians’ access to healthcare resources, whereas older adolescents may seek support beyond that provided by their families.

Earlier research highlighted the presence of a weight stigma in South Korea, a phenomenon not confined to adults but also evident among adolescents.^25^ Consequently, Korean adolescents may be subjected to sociocultural pressures regarding their appearance, exerted by their peers and parents. A previous study revealed that students in single-sex schools have a higher probability of having obesity than those in co-educational schools.^26^ Moreover, the incidence of disordered weight control behaviors is reportedly higher among girls, older adolescents, students at co-educational schools, and those from either high- or low-SES groups.^27^ These sociocultural pressures may partially account for the observed differences in age and sex in our study.

With respect to family dynamics during macroeconomic recessions, previous studies have suggested that economic distress during such times is associated with negative changes in parenting, resulting in reduced psychosocial well-being among adolescents.^28^ This phenomenon reportedly varies according to the sex of the parents, children, or adolescents. Under economic pressure, parents, especially fathers, become more punitive and uninvolved and less authoritative toward their children.^29^ Girls are reportedly more vulnerable to their father’s stress levels than boys, and sex-related differences in psychological results may in part be explained by mirroring of gender roles.^28^ External stressors often lead to hormonal and physical dysregulation in women; thus, psychological stress caused by family dynamics may result in obesity.^30^ Besides the family relationship perspective, decreased savings due to economic deterioration are also associated with impaired family functioning.^31^ Among Korean adolescents during the COVID-19 pandemic, research has demonstrated that perceived family-level financial deterioration is associated with worsened mental health, such as anxiety and suicidal ideation, among boys and especially among girls.^32^^–^^34^ Another study on Korean couples suggested that economic hardships due to COVID-19 impacted couple conflict through different pathways in each socioeconomic stratum.^35^ Family dynamics may partially explain the association between perceived financial insecurity and obesity, possibly contributing to age and sex differences.

To the best of our knowledge, this is the first study in which the association between perceived familial financial insecurity, as a combination of subjective SES and perceived family-level financial deterioration, with obesity was examined among adolescents during a macroeconomic recession. This study included a large, nationally representative sample, and missing data were minimized using the survey method.^14^ The availability of information on family-level financial deterioration is another strength of this study.

However, several limitations need to be addressed. First, all information on SES and financial deterioration was obtained through self-reported questionnaires. Although the reliability and validity of SES measures among adolescents are not extensively discussed and raise major concerns, it is reported that older adolescents’ perceptions of familial SES are more closely correlated with maternal perceptions than younger adolescents’ perception.^36^^,^^37^ The possible difference according to the age may have potentially affected the observed heterogeneity between the result of middle- and high-school students. In addition, subjective and objective SES in adolescence may have different aspects.^38^^,^^39^ It is suggested that subjective SES might offer a deeper insight into the individual’s relative position within their community, which can be a stronger determinant of their health outcomes, including obesity compared to objective measures.^37^^,^^40^ Conversely, potential mediating and reverse relationships have also been proposed between objective SES, subjective social status (SSS),^39^ and obesity. These include obesity mediating the pathway between SES and SSS, SSS mediating the pathway between SES and obesity, or obesity directly impacting SES. In our study, we utilized adolescent-reported SES, which potentially includes different aspects compared to those captured by objective SES. Therefore, the interpretation of the results requires careful consideration. Evidence on validity and potential misclassification of perceived financial deterioration is very limited. A study from Portugal during economic recession reported that having an unemployed parent might increase the perceived repercussions of the economic recession, while family wealth could mitigate them.^41^ To address this potential limitation, we conducted sensitivity analyses by re-categorizing subjective SES and perceived financial deterioration (data not shown). Reassuringly, our results remained consistent and robust across these different categorizations. Second, BMI was potentially underestimated because of overreporting of height and underreporting of weight. One study suggested the possibility that information on BMI in the KYRBS was underreported compared with that in the National Student Health Examination.^42^ Underreporting of BMI leads to an underestimation of the prevalence of obesity; however, this phenomenon is not likely to differ across financial insecurity levels. Hence, our results were likely not considerably influenced. Third, this study’s results may not be broadly applicable to typical macroeconomic recessions. The unique circumstances of the COVID-19 pandemic, including lockdown measures, drastically changed lifestyle factors, such as physical activity and eating habits. The extent to which the lockdown impacted health outcomes is still under investigation. However, the disparity in financial security may have widened the gap in access to a healthy lifestyle. This potential increase in lifestyle disparity could have strengthened the robustness of our results.^43^ Furthermore, increased family time at home might have facilitated smoother family communication or incited conflict. Thus, lifestyle changes and family dynamics during the COVID-19 pandemic could fundamentally differ from those experienced during a general macroeconomic recession. Fourth, since this study only encompassed data from 2020 to 2021, reflecting the initial phase of the COVID-19 pandemic, the results do not account for the subsequent social transitions and long-term impacts induced by the ongoing health and economic crisis. Our supplemental analyses of 10-year trend of obesity prevalence according to SES suggested that the disparity in obesity prevalence by subjective SES may have narrowed in boys during the early pandemic, attributed to a rise in prevalence among middle- and higher-SES boys, while widening in girls (as shown in eFigure 3). Considering the long-term increasing trend of obesity^4^ and the consistent SES disparity in obesity prevalence in Korean adolescents,^11^ it is crucial to carefully monitor long-term transitions including those occurring after lifting the lockdown period.

In this nationwide cross-sectional study of Korean adolescents, subjective household SES and perceived COVID-19-related financial deterioration were independently and jointly associated with obesity. Younger adolescents were more sensitive to household SES, whereas older adolescents were more sensitive to financial deterioration. The association between perceived financial insecurity and obesity was more prominent among girls. As a macroeconomic recession may aggravate socioeconomic disparity, special attention may be needed for adolescents from financially insecure families.

## SUPPLEMENTARY MATERIAL

eTable 1. General characteristics of participants

eTable 2. Association between household socioeconomic characteristics and obesity among adolescents

eTable 3. General characteristics of participants included in the exploratory analysis

eFigure 1. Flowchart of study participant selection

eFigure 2. Joint association of subjective household socioeconomic status and perceived COVID-19-related financial deterioration with obesity stratified by survey year

eFigure 3. 10-year trend of obesity prevalence by SES, KYRBS 2012–2021
