# Supplementary material for: Perceived Familial Financial Insecurity and Obesity Among Korean Adolescents During the COVID-19 Pandemic
Source: J Epidemiol. 2024 Dec 5;34(12):587–94. doi: 10.2188/jea.JE20240038 (PMC11564064; doi:10.2188/jea.JE20240038)
Supplement: Supplementary file 1 [file je-34-587-s001.pdf]

eTable 1. General characteristics of participants

Variables	Boys						Girls					
	Total (n=55,460)		2020 (n=27,687)		2021 (n=27,773)		Total (n=51,519)		2020 (n=25,847)		2021 (n=25,672)	
Age, years, mean (SD)	15.1	(1.7)	15.1	(1.8)	15.1	(1.7)	15.1	(1.8)	15.1	(1.8)	15.1	(1.8)
Obese, n (%)	9,369	(16.9)	4,435	(16.0)	4,934	(17.8)	4,638	(9.0)	2,244	(8.7)	2,394	(9.3)
School stage, n (%)												
Middle school	29,815	(53.8)	14,536	(52.5)	15,279	(55.0)	27,796	(54.0)	13,756	(53.2)	14,040	(54.7)
High school	25,645	(46.2)	13,151	(47.5)	12,494	(45.0)	23,723	(46.0)	12,091	(47.2)	11,632	(45.3)
Household socioeconomic status, n (%)												
High	6,888	(12.4)	3,434	(12.4)	3,454	(12.4)	4,717	(9.2)	2,428	(9.4)	2,289	(8.9)
Upper-middle	16,079	(29.0)	7,954	(28.7)	8,125	(29.3)	14,306	(27.8)	7,079	(27.4)	7,227	(28.2)
Middle	25,775	(46.5)	12,727	(46.0)	13,048	(47.0)	26,401	(51.3)	13,011	(50.3)	13,390	(52.2)
Lower-middle	5,449	(9.8)	2,902	(10.5)	2,547	(9.2)	5,165	(10.0)	2,822	(10.9)	2,343	(9.1)
Low	1,269	(2.3)	670	(2.4)	599	(2.2)	930	(1.8)	507	(2.0)	423	(1.7)
COVID-19-related financial deterioration, n (%)												
None	16,896	(30.5)	8,480	(30.6)	8,416	(30.3)	14,668	(28.5)	7,443	(28.8)	7,225	(28.1)
Minimal	21,525	(38.8)	10,775	(38.9)	10,750	(38.7)	21,476	(41.7)	10,575	(40.9)	10,901	(42.5)
Moderate	13,524	(24.4)	6,672	(24.1)	6,852	(24.7)	12,792	(24.8)	6,517	(25.2)	6,275	(24.4)
Severe	3,515	(6.3)	1,760	(6.4)	1,755	(6.3)	2,583	(5.0)	1,312	(5.1)	1,271	(5.0)
Urbanicity, n (%)												
Metropolitan city	24,307	(43.8)	12,268	(44.3)	12,039	(43.4)	22,091	(42.9)	10,790	(41.8)	11,301	(44.0)
Medium-sized or small-city	26,823	(48.4)	13,253	(47.9)	13,570	(48.9)	25,578	(49.7)	13,031	(50.4)	12,547	(48.9)
Rural area	4,330	(7.8)	2,166	(7.8)	2,164	(7.8)	3,850	(7.5)	2,026	(7.8)	1,824	(7.1)
Living arrangement, n (%)												
Living with family	52,745	(95.1)	26,284	(94.9)	26,461	(95.3)	49,476	(96.0)	24,764	(95.8)	24,712	(96.3)
Living with relatives	248	(0.5)	113	(0.4)	135	(0.5)	238	(0.5)	129	(0.5)	109	(0.4)
Other	2,467	(4.5)	1,290	(4.7)	1,177	(4.2)	1,805	(3.5)	954	(3.7)	851	(3.3)
School type, n (%)												
Co-educational school	32,920	(68.4)	18,592	(67.2)	19,328	(69.6)	34,156	(66.3)	17,054	(66.0)	17,102	(66.6)
All-male or all-female school	17,540	(31.6)	9,095	(32.9)	8,445	(30.4)	17,363	(33.7)	8,793	(34.0)	8,570	(33.4)

COVID-19, coronavirus disease 2019; SD, standard deviation.

eTable 2. Association between household socioeconomic characteristics and obesity among adolescents

Household socioeconomic characteristics	Boys				Girls			
	Number of adolescents	Number (%) of individuals with obesity	Odds ratio (95% CI)		Number of adolescents	Number (%) of individuals with obesity	Odds ratio (95% CI)	
			Model 1[a]	Model 2[b]			Model 1[a]	Model 2[b]
**Overall**								
Household socioeconomic status								
High	6,888	1,112 (16.1)	1.00 (Reference)	1.00 (Reference)	4,717	327 (6.9)	1.00 (Reference)	1.00 (Reference)
Upper-middle	16,079	2,614 (16.3)	1.01 (0.94–1.09)	1.00 (0.93–1.08)	14,306	1,062 (7.4)	1.01 (0.88–1.15)	0.99 (0.86–1.13)
Middle	25,755	4,363 (16.9)	1.06 (0.98–1.14)	1.03 (0.96–1.11)	26,401	2,415 (9.2)	1.20 (1.06–1.35)	1.12 (0.99–1.27)
Lower-middle	5,449	1,021 (18.7)	1.20 (1.10–1.31)	1.13 (1.03–1.24)	5,165	682 (13.2)	1.79 (1.55–2.06)	1.55 (1.34–1.80)
Low	1,269	259 (20.4)	1.35 (1.16–1.57)	1.26 (1.08–1.47)	930	152 (16.3)	2.28 (1.84–2.82)	1.88 (1.52–2.34)
COVID-19-related financial deterioration								
None	16,896	2,671 (15.8)	1.00 (Reference)	1.00 (Reference)	14,668	1,076 (7.3)	1.00 (Reference)	1.00 (Reference)
Minimal	21,525	3,568 (16.6)	1.05 (0.99–1.12)	1.04 (0.98–1.11)	21,476	1,847 (8.6)	1.14 (1.05–1.24)	1.09 (1.00–1.18)
Moderate	13,524	2,451 (18.1)	1.18 (1.11–1.26)	1.15 (1.08–1.23)	12,792	1,363 (10.7)	1.42 (1.30–1.55)	1.27 (1.16–1.39)
Severe	3,515	679 (19.3)	1.25 (1.13–1.38)	1.19 (1.07–1.32)	2,583	352 (13.6)	1.93 (1.69–2.20)	1.57 (1.36–1.80)
**Middle-school students**								
Household socioeconomic status								
High	4,378	693 (15.8)	1.00 (Reference)	1.00 (Reference)	3,237	185 (5.7)	1.00 (Reference)	1.00 (Reference)
Upper-middle	9,330	1,496 (16.0)	0.99 (0.89–1.09)	0.99 (0.89–1.09)	8,311	497 (6.0)	1.00 (0.93–1.20)	1.00 (0.83–1.19)
Middle	13,282	2,199 (16.6)	1.02 (0.93–1.13)	1.01 (0.92–1.12)	13,613	1,041 (7.7)	1.32 (1.11–1.56)	1.28 (1.08–1.53)
Lower-middle	2,361	434 (18.4)	1.21 (1.07–1.37)	1.17 (1.03–1.34)	2,260	228 (10.1)	1.77 (1.43–2.17)	1.60 (1.28–2.00)
Low	464	106 (22.8)	1.61 (1.27–2.03)	1.55 (1.22–1.96)	375	49 (13.1)	2.74 (1.95–3.84)	2.34 (1.63–3.36)
COVID-19-related financial deterioration								
None	9,464	1,505 (15.9)	1.00 (Reference)	1.00 (Reference)	8,540	543 (6.4)	1.00 (Reference)	1.00 (Reference)
Minimal	11,495	1,845 (16.1)	1.01 (0.93–1.09)	1.00 (0.92–1.09)	11,461	785 (6.9)	1.07 (0.96–1.19)	1.00 (0.89–1.12)
Moderate	7,074	1,241 (17.5)	1.10 (1.01–1.21)	1.07 (0.98–1.15)	6,560	540 (8.2)	1.31 (1.16–1.49)	1.16 (1.01–1.32)
Severe	1,782	337 (18.9)	1.19 (1.04–1.37)	1.12 (0.97–1.28)	1,235	132 (10.7)	1.81 (1.49–2.20)	1.44 (1.16–1.80)
**High-school students**								
Household socioeconomic status								
High	2,510	419 (16.7)	1.00 (Reference)	1.00 (Reference)	1,480	142 (9.6)	1.00 (Reference)	1.00 (Reference)
Upper-middle	6,749	1,118 (16.6)	1.03 (0.92–1.17)	1.02 (0.91–1.15)	5,995	565 (9.4)	0.99 (0.81–1.20)	0.96 (0.79–1.17)
Middle	12,493	2,164 (17.3)	1.10 (0.98–1.23)	1.06 (0.94–1.19)	12,788	1,374 (10.7)	1.10 (0.91–1.32)	0.99 (0.83–1.19)
Lower-middle	3,088	587 (19.0)	1.21 (1.06–1.38)	1.10 (0.96–1.26)	2,905	454 (15.6)	1.74 (1.42–2.13)	1.45 (1.18–1.78)
Low	805	153 (19.0)	1.26 (1.03–1.56)	1.14 (0.93–1.41)	555	103 (18.6)	2.03 (1.53–2.69)	1.62 (1.22–2.14)
COVID-19-related financial deterioration								
None	7,432	1,166 (15.7)	1.00 (Reference)	1.00 (Reference)	6,128	533 (8.7)	1.00 (Reference)	1.00 (Reference)
Minimal	10,030	1,723 (17.2)	1.11 (1.01–1.21)	1.09 (1.00–1.19)	10,015	1,062 (10.6)	1.21 (1.07–1.36)	1.17 (1.04–1.32)
Moderate	6,450	1,210 (18.8)	1.27 (1.16–1.39)	1.24 (1.13–1.36)	6,232	823 (13.2)	1.51 (1.34–1.70)	1.37 (1.21–1.56)
Severe	1,733	342 (19.7)	1.31 (1.23–1.52)	1.27 (1.09–1.48)	1,348	220 (16.3)	2.03 (1.70–2.42)	1.68 (1.40–2.02)

CI, confidence interval; COVID-19, coronavirus disease 2019.

[a]Model 1: Adjusted for age, living arrangement, and urbanicity

[b]Model 2: Adjusted for age, living arrangement, urbanicity, household socioeconomic status, and COVID-19-related financial deterioration

eTable 3. General characteristics of participants included in the exploratory analysis

Variables	Boys				Girls			
	Middle school (n=21,549)		High school (n=15,476)		Middle school (n=22,523)		High school (n=17,352)	
Age, years, mean (SD)	13.7	(0.9)	16.7	(0.9)	13.7	(0.9)	16.7	(0.9)
Obese, n (%)	3,620	(16.8)	2,740	(17.7)	1,589	(4.1)	1,949	(11.2)
Household socioeconomic status, n (%)								
Higher	10,281	(47.7)	5,811	(37.6)	9,704	(43.1)	5,725	(33.0)
Middle	9,470	(44.0)	7,557	(48.8)	10,898	(48.4)	9,355	(53.9)
Lower	1,798	(8.3)	2,108	(13.6)	1,921	(8.5)	2,272	(13.1)
COVID-19-related financial deterioration, n (%)								
None	7,027	(32.6)	4,639	(30.0)	950	(4.2)	4,607	(26.6)
Minimal	8,351	(38.8)	6,135	(39.6)	5,215	(23.2)	7,364	(42.4)
Moderate	5,016	(23.3)	3,791	(24.5)	9,252	(41.1)	4,462	(25.7)
Severe	1,155	(5.4)	911	(5.9)	7,106	(31.6)	919	(5.3)
Paternal educational attainment, n (%)								
University or higher	11,860	(55.0)	8,987	(58.1)	13,306	(59.1)	9,974	(57.5)
High school or lower	4,023	(18.7)	4,343	(28.1)	4,418	(19.6)	5,074	(29.2)
Not known	5,666	(26.3)	2,146	(13.9)	4,799	(21.3)	2,304	(13.3)
Maternal educational attainment, n (%)								
University or higher	11,793	(54.7)	8,592	(55.5)	13,179	(58.5)	9,523	(54.9)
High school or lower	4,295	(19.9)	4,745	(30.7)	5,344	(23.7)	6,031	(34.8)
Not known	5,461	(25.3)	2,139	(13.8)	4,000	(17.8)	1,798	(10.4)
Parental cohabitation, n (%)								
Father and mother	19,636	(91.1)	13,696	(88.5)	20,454	(90.8)	15,421	(88.9)
Only father	494	(2.3)	418	(2.7)	416	(1.9)	365	(2.1)
Only mother	1,271	(5.9)	1,119	(7.2)	1,515	(6.7)	1,353	(7.8)
Neither	148	(0.7)	243	(1.6)	138	(0.6)	213	(1.2)
Urbanicity, n (%)								
Metropolitan city	9,419	(43.7)	6,766	(43.7)	1,604	(7.1)	7,638	(44.0)
Medium-sized or small-city	10,631	(49.3)	7,389	(47.7)	9,598	(42.6)	8,386	(48.3)
Rural area	1,499	(7.0)	1,321	(8.5)	11,321	(50.3)	1,328	(7.7)
Living arrangement, n (%)								
Living with family	21,216	(98.5)	14,288	(92.3)	22,283	(98.9)	16,277	(93.8)
Living with relatives	58	(0.3)	46	(0.3)	62	(0.3)	57	(0.3)
Other	275	(1.3)	1,142	(7.4)	178	(0.8)	1,018	(5.9)
School type, n (%)								
Co-educational school	16,157	(75.0)	9,248	(59.8)	16,697	(74.1)	9,792	(56.4)
All-male or all-female school	5,392	(25.0)	6,228	(40.2)	5,826	(25.9)	7,560	(43.6)

COVID-19, coronavirus disease 2019; SD, standard deviation.

eFigure 1. Flowchart of study participant selection

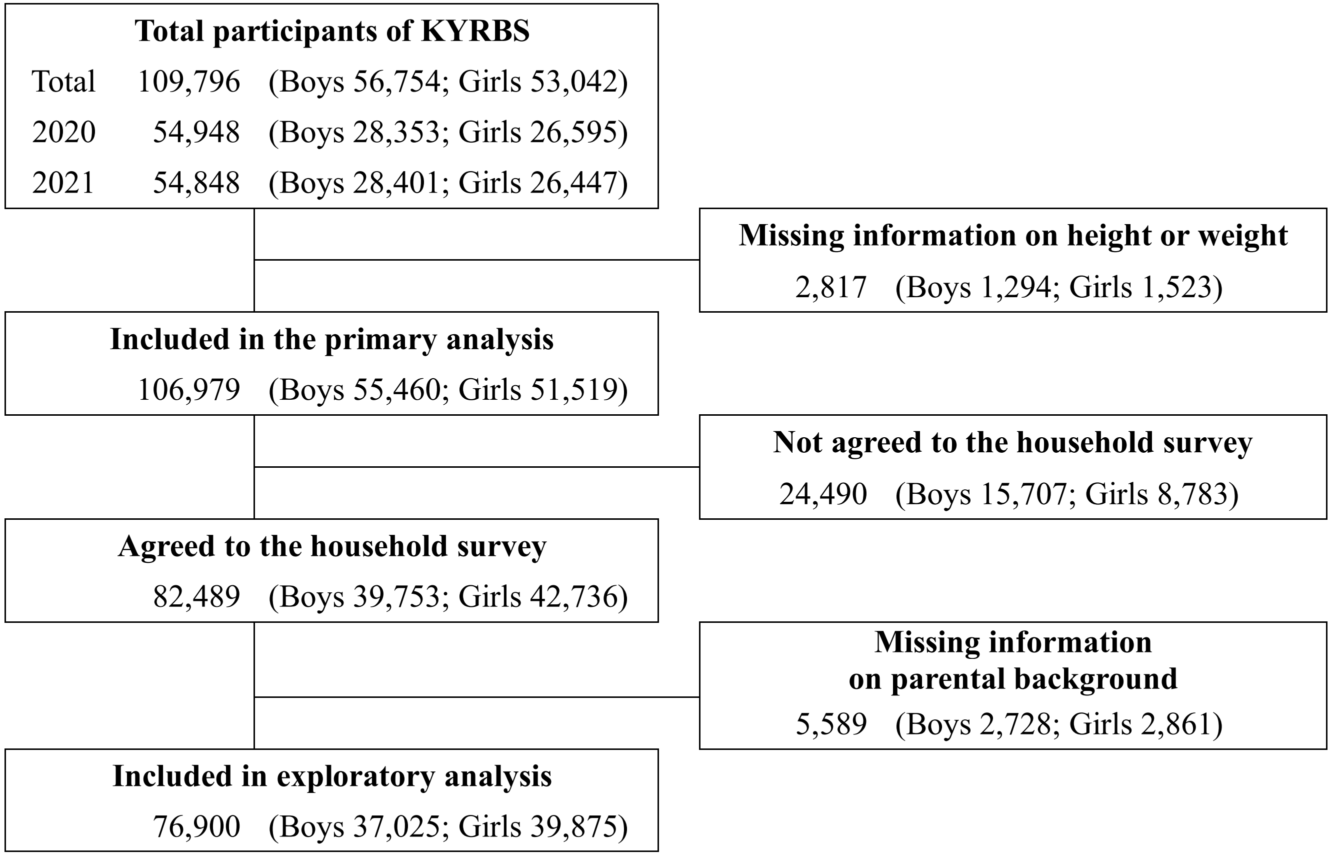

KYRBS, Korean Youth Risk Behavior Survey.

eFigure 2. Joint association of subjective household socioeconomic status and perceived COVID-19-related financial deterioration with obesity stratified by survey year

Boys

SES	Financial deterioration	2020 Number of adolescents	2020 Number (%) of obese	2020 Odds ratio (95% CI)	2021 Number of adolescents	2021 Number (%) of obese	2021 Odds ratio (95% CI)
**Overall**							
Higher	None	4992	706(14.1)	1.00 (Reference)	5080	838 (16.5)	1.00 (Reference)
	Minimal	3904	606(15.5)	1.08 (0.95–1.23)	4083	697 (17.1)	1.08 (0.95–1.21)
	Moderate	1925	328(17.0)	1.21 (1.04–1.41)	1923	359 (18.7)	1.16 (1.00–1.35)
	Severe	567	93(16.4)	1.14 (0.90–1.44)	493	99 (20.1)	1.09 (0.86–1.38)
Middle	None	3050	479(15.7)	1.08 (0.95–1.23)	3005	506 (16.8)	1.04 (0.92–1.18)
	Minimal	5681	871(15.3)	1.06 (0.94–1.19)	5740	1011 (17.6)	1.07 (0.97–1.19)
	Moderate	3393	586(17.3)	1.22 (1.08–1.38)	3676	680 (18.5)	1.20 (1.07–1.36)
	Severe	603	99(16.4)	1.24 (0.98–1.57)	627	131 (20.9)	1.36 (1.08–1.71)
Lower	None	438	88(20.1)	1.50 (1.18–1.89)	331	54 (16.3)	0.94 (0.69–1.27)
	Minimal	1190	211(17.7)	1.28 (1.08–1.53)	927	172 (18.6)	1.22 (1.00–1.48)
	Moderate	1354	262(19.4)	1.44 (1.22–1.70)	1253	236 (18.8)	1.17 (0.99–1.38)
	Severe	590	106(18.0)	1.27 (1.00–1.62)	635	151 (23.8)	1.55 (1.26–1.91)
**Middle-school students**							
Higher	None	2900	417	1.00 (Reference)	3076	509 (16.6)	1.00 (Reference)
	Minimal	2276	347	1.02 (0.86–1.21)	2452	410 (16.7)	1.02 (0.86–1.20)
	Moderate	1177	191	1.10 (0.90–1.34)	1193	208 (17.4)	1.09 (0.88–1.34)
	Severe	350	50	0.88 (0.64–1.19)	284	57 (20.1)	1.16 (0.83–1.62)
Middle	None	1539	253	1.09 (0.91–1.29)	1601	263 (16.4)	1.00 (0.85–1.17)
	Minimal	2852	408	0.94 (0.80–1.11)	3017	523 (17.3)	1.07 (0.94–1.23)
	Moderate	1691	290	1.08 (0.90–1.30)	1968	349 (17.7)	1.12 (0.95–1.33)
	Severe	294	45	1.07 (0.75–1.54)	320	68 (21.3)	1.33 (0.97–1.82)
Lower	None	192	39	1.69 (1.17–2.43)	156	24 (15.4)	0.88 (0.55–1.42)
	Minimal	500	91	1.34 (1.04–1.72)	398	66 (16.6)	1.17 (0.87–1.58)
	Moderate	504	106	1.56 (1.22–2.00)	541	97 (17.9)	1.02 (0.80–1.31)
	Severe	261	51	1.40 (0.98–1.98)	273	66 (24.2)	1.64 (1.18–2.28)
**High-school students**							
Higher	None	2092	289	1.00 (1.00–1.00)	2004	329 (16.4)	1.00 (Reference)
	Minimal	1628	259	1.16 (0.95–1.40)	1631	287 (17.6)	1.15 (0.97–1.37)
	Moderate	748	137	1.36 (1.08–1.72)	730	151 (20.7)	1.27 (1.03–1.56)
	Severe	217	43	1.55 (1.09–2.21)	209	42 (20.1)	1.04 (0.74–1.45)
Middle	None	1511	226	1.10 (0.91–1.34)	1404	243 (17.3)	1.10 (0.92–1.32)
	Minimal	2829	463	1.20 (1.00–1.43)	2723	488 (17.9)	1.09 (0.93–1.27)
	Moderate	1702	296	1.38 (1.16–1.64)	1708	331 (19.4)	1.29 (1.09–1.53)
	Severe	309	54	1.43 (1.04–1.96)	307	63 (20.5)	1.40 (1.01–1.96)
Lower	None	246	49	1.41 (1.03–1.93)	175	30 (17.1)	1.00 (0.66–1.49)
	Minimal	690	120	1.30 (1.01–1.67)	529	106 (20.0)	1.28 (0.98–1.65)
	Moderate	850	156	1.43 (1.15–1.80)	712	139 (19.5)	1.28 (1.03–1.61)
	Severe	329	55	1.22 (0.87–1.71)	362	85 (23.5)	1.54 (1.18–2.01)

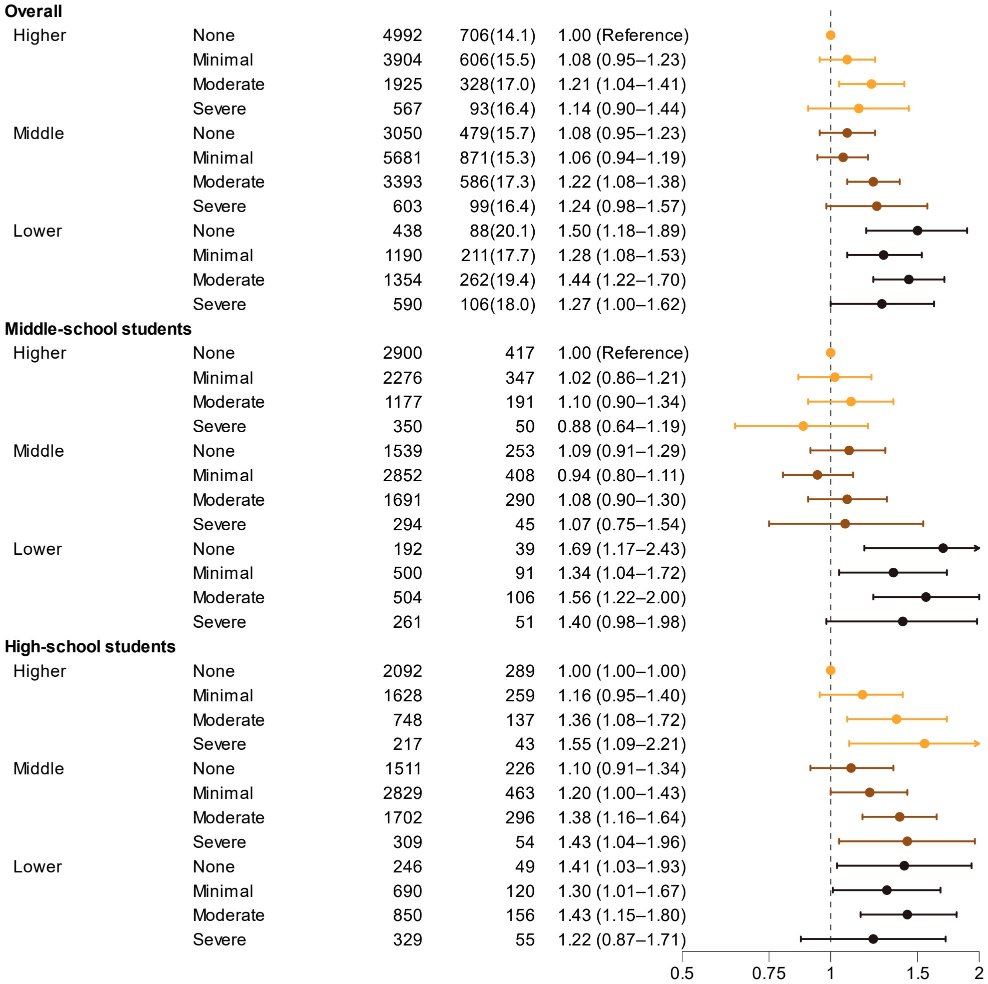

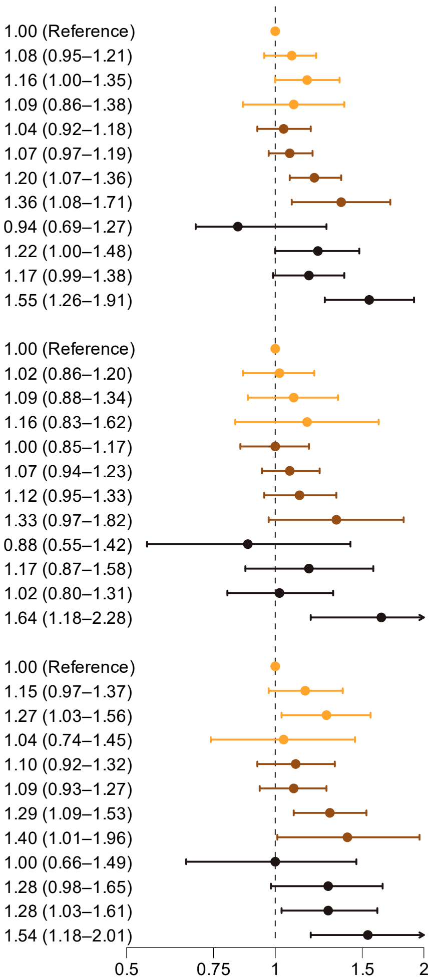

Girls

SES	Financial deterioration	2020 Number of adolescents	2020 Number (%) of obese	2020 Odds ratio (95% CI)	2021 Number of adolescents	2021 Number (%) of obese	2021 Odds ratio (95% CI)
**Overall**							
Higher	None	4284	296 (6.9)	1.00 (Reference)	4301	292 (6.8)	1.00 (Reference)
	Minimal	3521	229 (6.5)	1.08 (0.99–1.18)	3680	290 (7.9)	1.11 (0.93–1.31)
	Moderate	1467	110 (7.5)	1.05 (0.84–1.31)	1310	117 (8.9)	1.21 (0.96–1.51)
	Severe	235	23 (9.8)	1.58 (1.00–2.49)	225	32 (14.2)	1.95 (1.32–2.87)
Middle	None	2864	201 (7.0)	0.92 (0.75–1.12)	2742	231 (8.4)	1.12 (0.93–1.35)
	Minimal	6010	502 (8.4)	1.17 (1.00–1.37)	6386	596 (9.3)	1.23 (1.06–1.43)
	Moderate	3631	372 (10.3)	1.44 (1.21–1.71)	3731	393(10.5)	1.37 (1.17–1.61)
	Severe	506	58 (11.5)	1.55 (1.12–2.15)	531	62 (11.7)	1.57 (1.17–2.10)
Lower	None	295	34 (11.5)	1.41 (0.98–2.03)	182	22 (12.1)	1.54 (0.96–2.47)
	Minimal	1044	132 (12.6)	1.80 (1.47–2.21)	835	98 (11.7)	1.47 (1.15–1.87)
	Moderate	1419	197 (13.9)	2.00 (1.65–2.43)	1234	174 (14.1)	1.93 (1.55–2.41)
	Severe	571	90 (15.8)	2.34 (1.82–3.02)	515	87 (16.9)	2.68 (2.04–3.53)
**Middle-school students**							
Higher	None	2591	141 (5.4)	1.00 (Reference)	2681	160 (6.0)	1.00 (Reference)
	Minimal	2093	110 (5.3)	0.90 (0.69–1.18)	2212	133 (6.0)	1.00 (0.79–1.25)
	Moderate	877	48 (5.5)	0.90 (0.62–1.31)	817	58 (7.1)	1.12 (0.82–1.52)
	Severe	149	14 (9.4)	2.20 (1.23–3.92)	128	18 (14.1)	2.55 (1.56–4.14)
Middle	None	1516	94 (6.2)	1.11 (0.82–1.50)	1539	122 (7.9)	1.25 (0.96–1.62)
	Minimal	3015	217 (7.2)	1.42 (1.13–1.78)	3335	257 (7.7)	1.21 (0.99–1.48)
	Moderate	1823	158 (8.7)	1.70 (1.33–2.17)	1902	147 (7.7)	1.29 (1.02–1.63)
	Severe	241	21 (8.7)	1.36 (0.80–2.30)	242	25 (10.3)	1.69 (1.13–2.52)
Lower	None	126	13 (10.3)	2.35 (1.35–4.10)	87	13 (14.9)	2.84 (1.54–5.21)
	Minimal	451	39 (8.7)	1.49 (1.03–2.14)	355	29 (8.2)	1.26 (0.84–1.89)
	Moderate	624	73 (11.7)	2.30 (1.71–3.10)	517	56 (10.8)	1.90 (1.33–2.72)
	Severe	250	28 (11.2)	2.33 (1.49–3.66)	225	26 (11.6)	2.17 (1.37–3.42)
**High-school students**							
Higher	None	1693	155 (9.2)	1.00 (Reference)	1620	132 (8.2)	1.00 (Reference)
	Minimal	1428	119 (8.3)	0.87 (0.67–1.13)	1468	157 (10.7)	1.21 (0.94–1.55)
	Moderate	590	62 (10.5)	1.17 (0.88–1.55)	493	59 (12.0)	1.29 (0.93–1.79)
	Severe	86	9 (10.5)	1.08 (0.53–2.19)	97	14 (14.4)	1.46 (0.80–2.65)
Middle	None	1348	107 (7.9)	0.80 (0.61–1.04)	1203	109 (9.1)	1.02 (0.78–1.33)
	Minimal	2995	285 (9.5)	1.02 (0.82–1.26)	3051	339 (11.1)	1.24 (1.00–1.55)
	Moderate	1808	214 (11.8)	1.28 (1.00–1.63)	1829	246 (13.5)	1.43 (1.14–1.80)
	Severe	265	37 (14.0)	1.60 (1.05–2.43)	289	37 (12.8)	1.51 (1.01–2.27)
Lower	None	169	21 (12.4)	1.04 (0.65–1.64)	95	9 (9.5)	0.97 (0.47–2.02)
	Minimal	593	93 (15.7)	1.86 (1.44–2.40)	480	69 (14.4)	1.58 (1.15–2.17)
	Moderate	795	124 (15.6)	1.82 (1.41–2.35)	717	118 (16.5)	1.97 (1.48–2.62)
	Severe	321	62 (19.3)	2.29 (1.68–3.12)	290	61 (21.0)	2.97 (2.08–4.24)

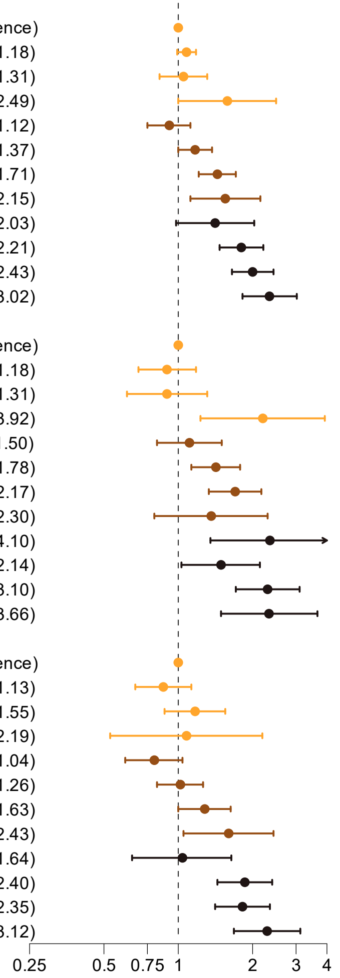

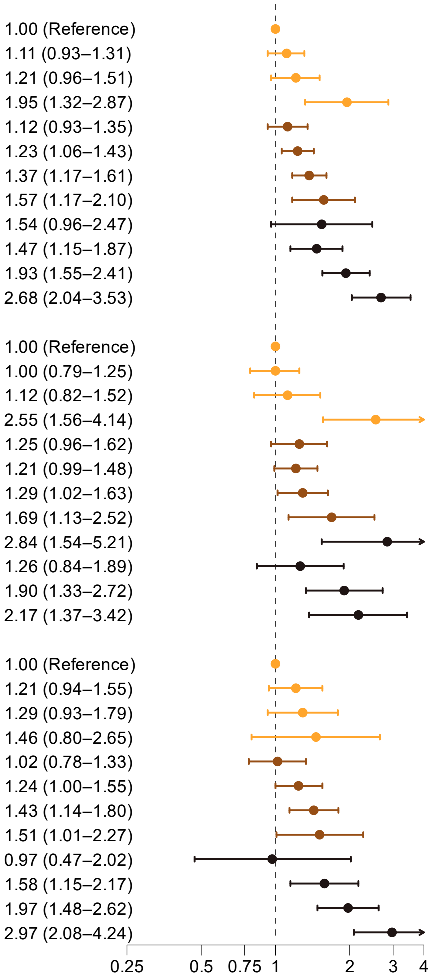

Adjusted for age, living arrangement, and urbanicity. CI, confidence interval; SES, socioeconomic status.

Girls

eFigure 3. 10-year trend of obesity prevalence by SES, KYRBS 2012–2021

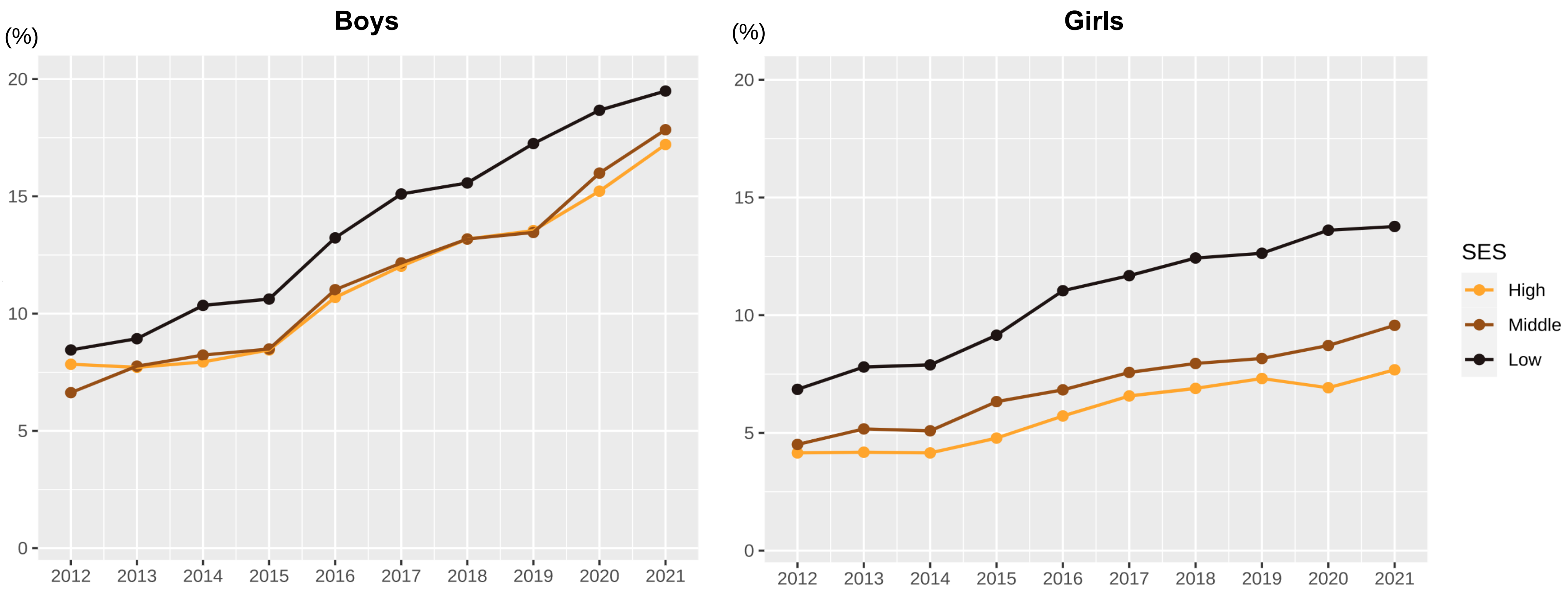

KYRBS, Korean Youth Risk Behavior Survey; SES, socioeconomic status.
